# The Right to Die

**Published:** 1900-04-28

**Authors:** 


### The Right to Die.

On the assumption that medical men hold the lives of their patients in the hollow of their hands, an assumption which, although complimentary to the powers of medicine, is not always consonant with truth, ingenious people not infrequently put forward curious problems as to the duty of the doctor under such responsible circumstances. Too often they blame him for not being careful enough of this precious possession, but now we are charged with holding on to life too firmly, and with interfering with man's "natural right to a natural death." In other words, complaint is made that doctors won't let people die. This is the text of an address recently delivered by the president of the American Social Science Association. He shows how the natural tendency of all the forces with which we are acquainted is towards a state of repose, that in the forces of the human body there is always decline and decay, and that life is a resistance to these processes of dissolution; that the moment surely comes when this resistance is overcome, and that when at last the organism has no answering process to meet the action of the outer world, that state of rest is reached with which life ends. This, then, is the "natural death" to which all people have "a natural right."

We are accustomed to regard unnatural death as something premature, but we are told "there is another form of unnatural death which we approach in the opposite direction. It is living too long." And it is against the unnatural death involved in this "living too long," which is put down to the credit, or, rather, to the discredit, of the doctors, that Dr. Baldwin raises his voice. He says: "Particularly is this true of the old man.

'For when his hand was palsied, and his eye was dim,
Then it was time to go.'

"Happy he if Nature calls him quietly away before the doctor can be sent for." In regard to this, we may, perhaps, suggest that, however truly it may be "time to go," not all old men are anxious so to do. But there are other cases. There are certain maladies which naturally end in a speedy death, but may be so treated, says Dr. Baldwin, as "to lead to a protracted state of weakness and suffering," incompatible with any enjoyment of life or useful activity, and from which there can be no real hope of ultimate recovery. In uncivilised nations such diseases are of short duration; they are either left to take their course without interference, or the patient is even expedited on his journey to the grave. In civilised nations, however, particularly of late years, "it has become the pride of many of the medical profession to prolong such lives at any cost of discomfort or pain to the sufferer, or of suspense and exhaustion to his family."

A vivid picture is drawn of the patient sinking from some incurable malady, "the vital forces have been spent. The mainspring is broken, and the watch has run down. It can be made to tick feebly for a minute or two by shaking it hard enough; but *cui bono?* . . . . The family ask the doctor if there is no hope, and he responds with some sharp stimulant; some hypodermic injection; some transfusion or infusion to fill out for a few hours the bloodless veins; some device for bringing oxygen into the congested lungs that cannot breathe the vital air; some cunning way of stimulating another organ to do the stomach's work. . . . The sufferer wakes to pain, and gasps back to a few more days or weeks of life," and, it is asked, "Were they worth the having?" Dr. Baldwin is by no means the first who has raised the question. Again and again, in the daily work of ordinary practice does one have to ask oneself, Is all this worth while? and again and again one has to decline to take the responsibility of saying "No." We do not say that no physician would be justified in standing aside and allowing Nature to take its course at the definite request of a patient still sound of mind. This is a free country, and no patient of full age and sound mind is bound to submit to any treatment which he does not like, however necessary to life it may appear, and no physician is responsible for the vagaries of such a patient even if they should prevent death being put off a little while. It is for the patient to decide, and we must confess that in regard to some great surgical triumphs the sort of life offered as the reward for the adventure is not such as to lead us to blame a man for preferring to exercise his "natural right to a natural death."

The difficulty comes when the patient is unconscious, or is an infant, or, perhaps, most hard case of all, where the patient in full faith and confidence says, "Doctor, I leave it to you." No duty ever thrown upon a medical man can be more trying than this of passing judgment upon one's patient. If a man accepts the responsibility of so doing he must first exercise the greatest diligence in making the diagnosis and prognosis sure, and then must exercise the greatest honesty in arriving at a conclusion. But there are many who may properly decline to accept, and there are some who are by nature unfitted to fill such a judicial position. Such people require rules of conduct, and to such the only safe rule is to hold on to life as long as a flicker lasts.

